# Identification of *p*-Coumaric Acid and Ethyl *p*-Coumarate as the Main Phenolic Components of Hemp (*Cannabis sativa* L.) Roots

**DOI:** 10.3390/molecules27092781

**Published:** 2022-04-27

**Authors:** Chang Min Oh, Joon Yong Choi, In Ah Bae, Hong Taek Kim, Seong Su Hong, Jay Kyun Noah, Yong Chool Boo

**Affiliations:** 1 Department of Biomedical Science, and BK21 Plus KNU Biomedical Convergence Program, The Graduate School, Kyungpook National University, 680 Gukchaebosang-ro, Jung-gu, Daegu 41944, Korea; ckdals9669@naver.com (C.M.O.); halo134679@naver.com (J.Y.C.); sksnadlv@naver.com (I.A.B.); aopp654@naver.com (H.T.K.); 2 Bio-Center, Gyeonggido Business & Science Accelerator (GBSA), 147 Gwanggyo-ro, Yeongtong-gu, Suwon-si 16229, Korea; bestgene@gbsa.or.kr; 3 Jayhempkorea, 211-13 Hyoja-ro, Hwaseo-myeon, Sangju-si 37142, Korea; jayk.noah@jayhempkorea.com; 4 Department of Molecular Medicine, and Cell and Matrix Research Institute, School of Medicine, Kyungpook National University, 680 Gukchaebosang-ro, Jung-gu, Daegu 41944, Korea

**Keywords:** hemp root, *Cannabis sativa*, *p*-coumaric acid, ethyl *p*-coumarate, phenolic compounds

## Abstract

Hemp (*Cannabis sativa* L.) contains a variety of secondary metabolites, including cannabinoids, such as psychoactive (−)-*trans*-Δ⁹-tetrahydrocannabinol. The present study was conducted to identify the major phenolic components contained in hemp root, which has been relatively under-researched compared to other parts of hemp. The aqueous ethanol extract of hemp roots was fractionated into methylene chloride (MC), ethyl acetate (EA), and water (WT) fractions, and high-performance liquid chromatography with photodiode array detection (HPLC-DAD) analysis was performed. The main ultraviolet (UV)-absorbing phenolic compound contained in the EA fraction was identified as *p*-coumaric acid by comparing the retention time and UV absorption spectrum with a standard. Silica gel column chromatography was performed to isolate a hydrophobic derivative of *p*-coumaric acid contained in the MC fraction. Nuclear magnetic resonance (NMR) analysis identified the isolated compound as ethyl *p*-coumarate. For comparative purposes, ethyl *p*-coumarate was also chemically synthesized by the esterification reaction of *p*-coumaric acid. The content of *p*-coumaric acid and ethyl *p*-coumarate in the total extract of hemp root was estimated to be 2.61 mg g^−1^ and 6.47 mg g^−1^, respectively, by HPLC-DAD analysis. These values correspond to 84 mg Kg^−1^ dry root and 216 mg Kg^−^^1^ dry root, respectively. In conclusion, this study identified *p*-coumaric acid and ethyl *p*-coumarate as the main phenolic compounds contained in the hemp roots.

## 1. Introduction

Hemp (*Cannabis sativa* L.) is an annual herbaceous plant belonging to the *Cannabaceae* family. Its origin is Central Asia, and various varieties are cultivated around the world, including in Canada, the United States, and Europe [1]. Hemp varieties commonly contain several cannabinoids and are a source of various secondary metabolites, such as terpenoids, flavonoids, and phytosterols [2]. Among the cannabinoids in hemp, (−)-*trans*-Δ⁹-tetrahydrocannabinol (THC), a known psychoactive substance, and cannabidiol (CBD), a non-psychoactive but highly physiologically active substance, are represented [3,4]. THC has anti-inflammatory, anti-nociceptive, detoxifying, and muscle relaxant properties, while CBD has antioxidant, anti-inflammatory, and immunomodulatory properties [4,5]. Hemp is divided into medical and industrial uses according to the THC content, and the THC content of industrial hemp is usually limited to 0.3% or less [3].

Hemp is a crop that has been traditionally grown for a long time to obtain fibers and seeds. Its application fields have recently been expanded to include agriculture, textiles, food, biofuel, cosmetics, and pharmaceutical industries [2,6]. Different parts of the hemp plant are used in a variety of ways. Hemp stems are used to produce fibers in the textile and paper industry [7,8]. Hemp wastes are used as raw materials for biofuel and cement replacement [9]. Hemp seeds are used as food and nutritional supplements for essential amino acids, proteins, and polyunsaturated fatty acids [10], and hemp seed oil also contains lignanamides and polyphenolic compounds with biological activities [11,12].

Hemp roots have been prescribed in traditional medicine to treat inflammation, joint pain, gout, and other pathological conditions [13]. Although there have been relatively few studies on the pharmacology of the roots compared to the flowers or leaves of cannabis species, studies on its pharmacological activity have recently increased in number. Lima et al. [14] evaluated the anti-inflammatory and spasmolytic effects and the single-dose and repeated-dose toxicity of the aqueous extract of hemp roots in vitro, ex vivo, and in vivo in mice. The results demonstrated the anti-inflammatory effects of the hemp root extract with no spasmolytic effect on airway smooth muscle and no notable toxicity in mice. Menezes et al. [15] suggested that hemp root water extract has the potential to treat cough and promote fluid expectoration in an animal study using male mice.

Hemp roots contain various biologically active phytochemicals. The compounds isolated from hemp roots include friedelin, friedelan-3-one, epifriedelinol (also called epifriedelanol), β-sitosterol, β-sitosterol-β-D-glucoside, ergost-5-en-3-ol, carvone, dihydrocarvone, methyl hexadecanoate, pentadecanoic acid, 10E-hexadecenoic acid, 4-hydroxy-3-methoxybenzaldehyde, and *N*-(*p*-hydroxy-*β*-phenylethyl)-*p*-hydroxy-*trans*-cinnamamide (also called *p*-coumaroyltyramine) [16,17,18]. A recent study reported that various cannabinoids, such as THC and CBD, could even be detected in roots using liquid chromatography (LC)–tandem mass spectrometry (MS) [19]. However, compared to the aerial parts of hemp, the content of cannabinoids in the roots is negligible, while triterpenes and phytosterols are present at high concentrations: Five triterpenes, ten phytosterols, and five aliphatic compounds were identified by gas chromatography (GC)–MS analysis [20]. Menezes et al. [15] identified *p*-coumaroyltyramine, tetrahydrocannabinol-C4, feruloyltyramine, anhydrocanabisativine, and cannabisativine in hemp roots using LC–MS.

The present study was conducted to identify the main phenolic compounds contained in the roots of hemp grown in Korea. As a result, it was found that *p*-coumaric acid and ethyl *p*-coumarate were contained in high concentrations in the hemp roots. In particular, ethyl *p*-coumarate was isolated from hemp roots for the first time.

## 2. Materials and Methods

### 2.1. Reagents

*p*-Coumaric acid (Cat. C9008), tetramethylsilane (TMS) (cat. T24007), and deuterochloroform (CDCl_3_) (cat. 151823) were purchased from Sigma-Aldrich (St. Louis, MO, USA). Ethyl *p*-coumarate (Cat. A104956) was purchased from Ambeed Inc. (Arlington Heights, IL, USA).

### 2.2. Preparation of the Total Extract of Hemp Roots and Its Solvent Fractions

Hemp seeds (*Cannabis sativa* L.; cultivar name, cheungsam) were obtained from Dr. Youn-Ho Moon, National Institute of Crop Science, Rural Development Administration, Korea (Muan-gun, Jeollanam-do, Korea), and cultivated by Jayhempkorea (Sangju-si, Gyeongsangbuk-do, Korea). After harvesting the above-ground parts of hemp grown in Sangju-si (Gyeongsangbuk-do, Korea) in October 2022, the remaining roots were collected and used in this study. Hemp roots were washed with water, air-dried in the shade, chopped, and ground in a blender. For extraction, 400 g of dry powder of hemp roots and 4 L of 70% aqueous ethanol were added to a glass bottle, mixed well, and stored for 14 days with occasional mixing in the shade at a room temperature of about 25 °C. The plant residue and the filtrate were separated using Corning 500 mL bottle-top vacuum filter (Cat. 431118) (Corning, Corning, NY, USA) and the filtrate was concentrated to dryness under reduced pressure using a rotary evaporator (Eyela, Bohemia, NY, USA) to obtain 12.8 g of a total extract of hemp roots. The total extract was dispersed in 200 mL of water (WT) and put in a separation funnel; subsequently, an equal volume of methylene chloride (MC) was added, mixed well, and partitioned into an MC layer and an aqueous layer. After collecting the MC layer in a separate container, 200 mL of ethyl acetate (EA) was added to the separating funnel, mixed with the aqueous layer, and divided into an EA layer and an aqueous layer. Each layer was concentrated to dryness under reduced pressure using a rotary evaporator to prepare 4.4 g of MC fraction, 0.24 g of EA fraction, and 8.24 g of WT fraction.

### 2.3. High-Performance Liquid Chromatography with Photodiode Array Detection (HPLC-DAD)

HPLC-DAD analysis was performed as described previously [21]. Waters Alliance HPLC system (Waters, Milford, MA, USA) consists of an e2695 separation module and a 2996 photodiode array detector (DAD). The stationary phase used is a Hector-M C_18_ column (4.6 mm × 250 mm, 5 μm) (RS Tech Co., Daejeon, Korea). The mobile phase used is a mixture of 0.1% phosphoric acid (solvent A) and acetonitrile (solvent B) with a variable composition: 0–30 min, a linear gradient from 0 to 100% B; 30–40 min, 100% B; 40–45 min, a linear gradient from 100 to 0% B. The mobile phase was eluted at a flow rate of 0.6 mL min^−1^ and a 10 μL volume of the sample was injected. The content of *p*-coumaric acid and ethyl *p*-coumarate in the total extract or each fraction was determined by extrapolation from the calibration curves (peak height versus concentration) prepared for each standard and are expressed in units of mg g^−1^.

### 2.4. Isolation of a Phenolic Compound from the MC Fraction

Silica gel column chromatography was performed to separate the substance presumed to be a hydrophobic derivative of *p*-coumaric acid contained in the MC fraction. First, 2 g of the MC fraction was loaded into a column (ϕ3 cm × 20 cm) filled with silica gel (Cat. 288624, Sigma-Aldrich), and then eluted with a 99:1 (*v*/*v*) mixed solvent of MC and methanol. The eluted fractions were subjected to silica gel thin-layer chromatography to examine whether or not the target material was contained, and the fractions containing the target material were combined and evaporated under reduced pressure to obtain 0.21 g of a purified substance. For the second silica gel column chromatographic separation of this substance, it was loaded onto a silica gel column and eluted with a mixed solvent of hexane and EA with a stepwise gradient from 6:1 to 4:1 (*v*/*v*). The eluted fractions were subjected to silica gel thin-layer chromatography, and the fractions containing the target material were combined and evaporated under reduced pressure to obtain 18 mg of Compound A.

### 2.5. Nuclear Magnetic Resonance (NMR) Spectroscopy

Proton [^1^H] and carbon 13 [^13^C]-NMR spectra were measured using a Bruker Ascend III 700 spectrometer (Bruker BioSpin, Rheinstetten, Germany). TMS was used as an internal standard, and chemical shifts were expressed as δ values. ^1^H-^1^H COSY (correlated spectroscopy), HSQC (heteronuclear single quantum coherence spectroscopy), and HMBC (heteronuclear multiple bond correlation) experiments were performed to assign the signals to the atoms of the compound and the interactions between them.

### 2.6. Chemical Synthesis of Ethyl p-Coumarate

Ethyl *p*-coumarate was prepared by esterification of *p*-coumaric acid [22]. *p*-Coumaric acid (100 mg, 0.61 mmol) was dissolved in ethanol (10 mL, 170 mmol), sulfuric acid (0.1 mL, 1.9 mmol) was added, and reacted at 70 °C under reflux for 5 h. Silica gel thin-layer chromatography was performed using a 6:1 (*v*/*v*) mixed solvent of hexane and EA to confirm the completion of the reaction. To the reaction mixture, 5% aqueous NaHCO_3_ solution (ca. 4.1 mL) was added to neutralize to the solution to pH 7.0, and it was then evaporated to dryness under reduced pressure. EA (10 mL) and water (10 mL) were added to the dry material and vigorously mixed to dissolve it, and the mixture was then divided into EA and aqueous layers using a separating funnel. The EA layer was evaporated under reduced pressure to dryness to obtain ethyl *p*-coumarate (64 mg, 0.33 mmol) as a crystalline powder.

### 2.7. Ultraviolet (UV) Spectroscopy

The UV absorption spectra of the sample in methanol were recorded with a Shimadzu UV-1650PC spectrophotometer (Shimadzu Corporation, Kyoto, Japan) in the range of 220–400 nm.

### 2.8. Statistical Analysis

The experimental results are presented as the mean ± standard deviation (SD). SigmaStat v.3.11 (Systat Software Inc., San Jose, CA, USA) was used for the statistical analysis of the data. One-way analysis of variance (ANOVA) was performed to determine the existence of differences in group means at *p* < 0.05 level. Duncan’s multiple range test was used to compare all experimental groups to each other.

## 3. Results

### 3.1. Preparation of the Total Extract of Hemp Roots and Its Solvent Fractions

**Figure 1:**
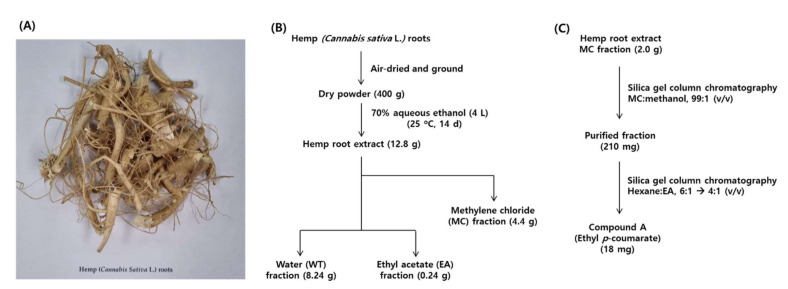
Preparation of hemp (*Cannabis sativa* L.) roots-derived materials used in this study. (**A**) A photograph of the hemp roots. (**B**) A procedure for the preparation of the total extract of hemp roots and its solvent fractions. The total extract was separated into methylene chloride (MC), ethyl acetate (EA), and water (WT) fractions. (**C**) Separation of compound A (ethyl *p*-coumarate) from the MC fraction by two successive silica gel column chromatographic separations using different eluting solvents.

Hemp roots used in this study are shown in Figure 1A. The total extract of hemp roots and its solvent fractions were prepared according to the procedure shown in Figure 1B. 

### 3.2. Identification of p-Coumaric Acid Contained in the EA Fraction

**Figure 2:**
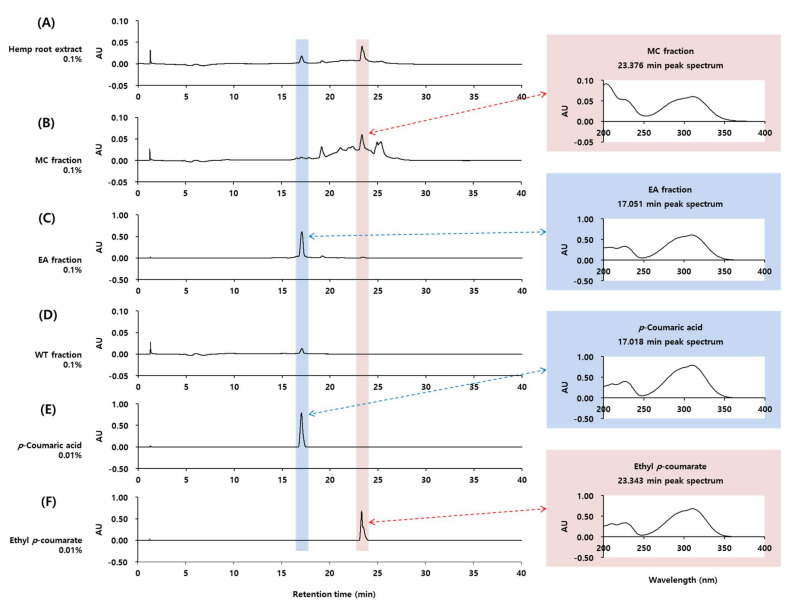
High-performance liquid chromatography with photodiode array detection (HPLC-DAD) analysis of the total extract of hemp roots (**A**) and its MC fraction (**B**), EA fraction (**C**), and WT fraction (**D**). *p*-Coumaric acid (**E**) and ethyl *p*-coumarate (**F**) were compared for retention times and absorption spectra. Chromatograms detected at 310 nm and UV absorption spectra of the indicated peaks are shown.

To obtain information on the phytochemicals contained in the total extract of hemp roots and its solvent fractions, HPLC-DAD analysis was performed; the results are shown in Figure 2. First, the main peak observed in the EA fraction was predicted to be *p*-coumaric acid from its UV absorption spectrum. By comparing the retention time and UV absorption spectrum of authentic *p*-coumaric acid, it was confirmed that this peak corresponds to *p*-coumaric acid.

### 3.3. Isolation and Identification of Ethyl p-Coumarate Contained in the MC Fraction

A peak showing a UV absorption spectrum similar to that of *p*-coumaric acid was observed in the MC fraction, and it was assumed to result from a hydrophobic derivative of *p*-coumaric acid. Following to the process illustrated in Figure 1C, the target compound was separated from the MC fraction through two consecutive column chromatography using different solvents. 

^1^H- and ^13^C-NMR analyses of the isolated compound were performed (Table 1). This compound was identified as ethyl *p*-coumarate by comparison with the data reported in the literature [22]. Thus, one of the main compounds in the MC fraction of hemp root extract was identified to be ethyl *p*-coumarate.

### 3.4. Chemical Synthesis of Ethyl p-Coumarate

**Figure 3:**
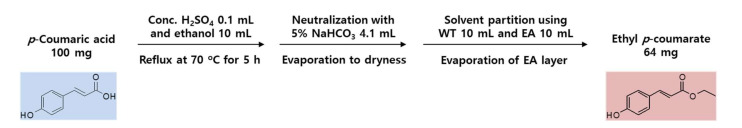
Chemical synthesis of ethyl *p*-coumarate from *p*-coumaric acid.

For comparative purposes, ethyl *p*-coumarate was chemically synthesized by esterification of *p*-coumaric acid and purified according to the procedure illustrated in Figure 3. The compound isolated from the hemp roots and the chemically synthesized compound were confirmed to be identical based on the HPLC-DAD analysis.

### 3.5. UV Absorption Spectra of the Hemp Root Extract and Its Components

**Figure 4:**
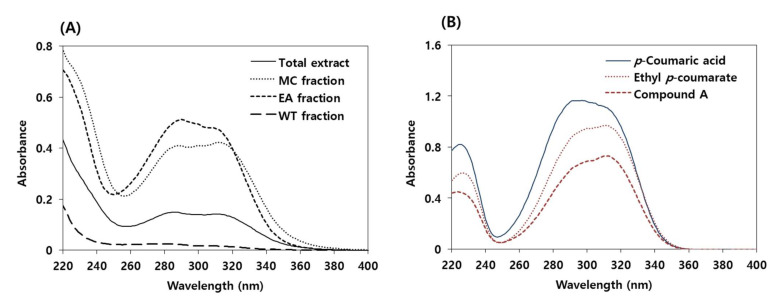
Ultraviolet (UV) absorption spectra of the samples in methanol. (**A**) UV absorption spectra of the total hemp root extract, its MC fraction, EA fraction, and WT fraction (30 μg mL^−1^). (**B**) UV absorption spectra of *p*-coumaric acid and ethyl *p*-coumarate chemically synthesized from *p*-coumaric acid (10 μg mL^−1^). The UV absorption spectrum of compound A purified from the hemp roots is also shown for comparison.

In Figure 4, their UV absorption spectra are compared with each other and with that of *p*-coumaric acid. In addition, the UV absorption spectra of the total extract of hemp root and each solvent fraction were compared. The UV absorption spectra of *p*-coumaric acid and ethyl *p*-coumarate in methanol solution had different maximum absorption wavelengths: 292 nm and 312 nm, respectively. Interestingly, the EA fraction and its main component, *p*-coumaric acid, had similar absorption spectra. In addition, the absorption spectrum of the MC fraction was similar to that of its main component, ethyl *p*-coumarate.

### 3.6. Content of p-Coumaric Acid and Ethyl p-Coumarate in the Hemp Root Extract and Its Solvent Fractions

The content of *p*-coumaric acid and ethyl *p*-coumarate in the total extract of hemp root and each solvent fraction was determined through HPLC-DAD analysis; the results are shown in Table 2. The content of *p*-coumaric acid and ethyl *p*-coumarate in the total extract was 2.61 mg g^−1^ and 6.47 mg g^−1^, respectively; the content of the latter was higher. *p*-Coumaric acid content was highest in the EA fraction (83.37 mg g^−1^), followed by the WT fraction (1.66 mg g^−1^) and the MC fraction (0.46 mg g^−1^). Ethyl *p*-coumarate content was highest in the MC fraction (17.25 mg g^−1^), followed by the EA fraction (3.32 mg g^−1^).

## 4. Discussion

Izzo et al. [23] analyzed the content of various phenolic compounds of four different commercial cultivars of hemp inflorescences using ultra-high-performance liquid chromatography and Q-exactive Orbitrap high-resolution mass spectrometry (UHPLC-Q-Orbitrap HRMS): hydroxycinnamic acids, such as caffeic acid, *p*-coumaric acid, and ferulic acid, and chlorogenic acid; lignanamides, such as cannabisin A, cannabisin B, and cannabisin C; phenolic amides, such as caffeoyltyramine; flavonoids, such as quercetin, kaempferol, cannflavin A, cannflavin B, luteolin, apigenin, catechin, epicatechin, naringenin, rutin, quercetin-3-*O*-glucoside, kaempferol-3-*O*-glucoside, luteolin-7-*O*-glucoside, and apigenin-7-*O*-glucoside. Jin et al. [24] comprehensively profiled secondary metabolites in hemp plants, including 14 cannabinoids, 47 terpenoids, 3 sterols, and 7 flavonoids in flowers, leaves, stem barks, and roots of three chemotypes of cannabis [25]. They also compared the full spectrum of secondary metabolites in THC-dominant, CBD-dominant, and intermediate strains. The identified chemotype markers would be useful for quality standardization in research and industry.

Our present study demonstrated that the extract of hemp roots contained a high concentration of *p*-coumaric acid. *p*-Coumaric acid is a representative phenylpropanoid compound widely distributed in the plant kingdom [26]; the compound and its derivatives have antioxidant, antimicrobial, anti-inflammatory, and various other biological activities [26,27]. *p*-Coumaric acid effectively scavenges free radicals [28] and relieves oxidative stress and inflammatory response of endothelial cells and epidermal keratinocytes [29,30]. It also attenuates alcohol hepatotoxicity and protects the skin from UV radiation [31,32]. Additionally, *p*-coumaric acid inhibits the activity of tyrosinase by a competitive mechanism with the substrate [33,34] and reduces pigmentation in animal models and human application tests [35,36]. Therefore, *p*-coumaric acid is being developed for various uses such as active ingredients in cosmetics [37].

It was reported in the current study for the first time that ethyl *p*-coumarate was contained in hemp root. Ethyl *p*-coumarate was shown to exhibit pronounced antifungal activity against *Alternaria alternata* in vitro and in vivo in jujube fruit, suggesting its potential to be used in controlling postharvest disease in fruits [38]. Its antioxidant and anti-inflammatory effects were demonstrated in the carrageenan-induced paw edema model of Swiss mice and Freund’s complete adjuvant-induced arthritis model of Wistar rats [39]. Recently, Li et al. [40] isolated ethyl *p*-coumarate from camellia pollen and reported its inhibitory action on mushroom tyrosinase.

In conclusion, this study identified *p*-coumaric acid and ethyl *p*-coumarate as the main phenolic compounds contained in the hemp roots. The content of *p*-coumaric acid and ethyl *p*-coumarate were 84 mg Kg^−^^1^ dry root and 216 mg Kg^−^^1^ dry root, respectively. The information would be useful in the research and industrial application of hemp roots. Further studies are needed to examine the biological activities of the hemp roots-derived phytochemicals.

## Figures and Tables

**Table 1:** ^1^H- and ^13^C-nuclear magnetic resonance (NMR) spectroscopic data of purified compound A (ethyl *p*-coumarate).

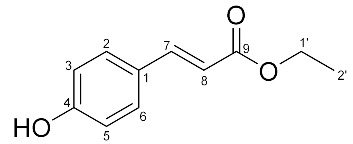			
**Position**	**δ_H_ Multiplicity (*J* Hz)**	**δ_C_ Multiplicity**	**HMBC**
1	-	127.0 s	-
2	7.42 d (8.4)	130.0 d	1, 3, 4, 5, 6, 7
3	6.86 d (8.4)	115.9 d	1, 4, 5
4	-	158.0 s	-
5	6.86 d (8.4)	115.9 d	1, 3, 4
6	7.42 d (8.4)	130.0 d	1, 2, 3, 4, 5, 7
7	7.64 d (16.1)	144.7 d	1, 2, 6, 8, 9
8	6.30 d (16.1)	115.4 d	1, 9
9	-	167.9 s	-
1′	4.27 q (7.0)	60.6 t	2′, 9
2′	1.34 t (7.0)	14.3 q	1′

Measured at 700 and 175 MHz; obtained in CDCl_3_ with TMS as an internal standard. The assignments were confirmed by ^1^H-^1^H COSY (correlated spectroscopy), HSQC (heteronuclear single quantum coherence spectroscopy), and HMBC (heteronuclear multiple bond correlation) experiments. Multiplicity: s, singlet; d, doublet; t, triplet; q, quintet.

**Table 2:** Content of *p*-coumaric acid and ethyl *p*-coumarate in the total extract of hemp roots and its solvent fractions.

Samples	Content (mg g^−1^)	
	*p*-Coumaric Acid	Ethyl *p*-Coumarate
Total extract	2.61 ± 0.18 ^b^	6.47 ± 0.95 ^b^
MC fraction	0.46 ± 0.20 ^b^	17.25 ± 3.04 ^a^
EA fraction	83.37 ± 2.44 ^a^	3.32 ± 0.29 ^c^
WT fraction	1.66 ± 0.09 ^b^	0 ± 0 ^d^

The data from HPLC-DAD analysis are presented as mean ± SD (*n* = 3). Duncan’s multiple range test was used in comparing all group means to each other. Different letters (a–d) in each column indicate significantly different means at the *p* < 0.05 level.

## Data Availability

Not applicable.
